# A classroom intervention targeting working memory, attention and language skills: a cluster randomised feasibility trial

**DOI:** 10.1186/s40814-021-00771-w

**Published:** 2021-02-06

**Authors:** Anita Rowe, Jill Titterington, Joni Holmes, Lucy Henry, Laurence Taggart

**Affiliations:** 1 Institute of Nursing and Health Research, Ulster University, Shore Road, Newtownabbey, Co Antrim, BT37 0QB Northern Ireland; 2 MRC Cognition & Brain Sciences Unit, University of Cambridge, 15 Chaucer Road, Cambridge, CB2 7EF England; 3 Division of Language and Communication Science, City University of London, 10 Northampton Square, London, EC1V 0HB England

**Keywords:** Working memory, Classroom interventions, Dosage, Working memory, Attention, Language, Feasibility

## Abstract

**Background:**

International debate around the best models of speech and language therapy provision for children with language disorders has highlighted the need for research into classroom-based approaches and intervention dosage. Working memory (WM) is a cognitive skill linked to attention and language. ‘Recall to Enhance Children’s Attention, Language and Learning’ (RECALL) is a novel, 6-week, classroom-based intervention delivered by health professionals (HPs) and teachers. It is designed to target WM and enhance attention and language skills in 4–5 year olds.

**Methods:**

A cluster randomised feasibility trial was conducted to investigate aspects of the feasibility of a definitive trial to evaluate RECALL: (i) recruitment and sampling procedures; (ii) compliance and fidelity; (iii) the acceptability of RECALL to HPs and teachers; (iv) the appropriateness of the outcome measures. Six classes of 4–5 year olds participated: two received RECALL, two received an existing intervention targeting attention skills (not underpinned by WM theory), and two received education as usual (no intervention). Ten children in each class (*n =* 60) were sampled to assess the appropriateness of the outcome measures. Classroom observations were conducted to measure fidelity and semi-structured interviews with HPs, and teachers explored the acceptability of RECALL.

**Results:**

The recruitment targets were met, and all six schools completed the trial, but the sampling procedures require modification. Compliance was good (95% of RECALL sessions were delivered), but fidelity to the intervention protocol varied between 76% and 45% across the two schools. This was influenced by large class sizes, child factors, and facilitator factors, e.g., their understanding of the theory underpinning the intervention. The lack of fidelity reduced the dose (number of practice items) accessed by individual children, particularly those most at risk. There were mixed findings regarding the acceptability of RECALL and the appropriateness of the outcome measures.

**Conclusions:**

The trial protocol could be easily scaled-up in a future definitive trial, with an amended sampling procedure. RECALL should be repackaged as a small group intervention to enhance the fidelity of its delivery and its acceptability to HPs and teachers. This study highlights the need for thorough training for professionals who deliver classroom-based interventions for children with language disorders.

**Trial registration:**

ISRCTN13633886. Registered on 7 September 2018

**Supplementary Information:**

The online version contains supplementary material available at 10.1186/s40814-021-00771-w.

## Key messages regarding feasibility

- This study addressed the acceptability of a novel intervention for children with language disorders and the feasibility of delivering it to whole classes of 4–5-year-old children.
- The fidelity of the intervention delivery was compromised by large class sizes, child factors and facilitator factors, which could ultimately reduce the dose and potency of the intervention.
- The fidelity of the intervention delivery could be optimised if it were repackaged as a small group intervention, supported by direct training for the professionals who would deliver it in the classroom.

## Background

### The use of classroom-based interventions

Worldwide, there has been debate around the best models of Speech and Language Therapy (SLT) provision for school-aged children who are at risk of language disorders, particularly those from areas of social disadvantage (SD) where high proportions of children present with impoverished language skills on school entry [1, 2]. SLT services are increasingly providing collaborative, classroom-based interventions, but there is a lack of research-based evidence for this approach [3]. This raises important questions about whether valuable and limited resources are being used in the most efficient way [3–5]. Due to the role SLTs have in early intervention and prevention for language disorders, there is a need for ecologically valid research (conducted in real-life contexts) to provide an evidence-based practice approach [6, 7].

### Context of the current study

The current study was conducted in the real-life context of health and education services in one region of the UK, Northern Ireland (NI), where there are high rates of SD associated with educational underachievement [8, 9]. Extending the role of health professionals (HPs) within early intervention and integrated service provision across the health and education sectors is a key strategy [10] that aims to harness HPs' specialist knowledge of the developmental skills that form the foundation for learning (e.g., language and motor skills) to enhance educational practice. The Regional Integrated Support for Education (RISE) teams are based in five Health and Social Care Trusts (HSCTs) that provide integrated health and social care services across NI. The RISE teams include speech and language therapists (SLTs), occupational therapists (OTs), physiotherapists (PTs) and social, emotional and behavioural specialists (SEBs). They provide individualised (specialist) support for children referred by their teachers and whole class (targeted and universal) interventions that aim to prevent potential future difficulties for at risk children (i.e., non-referred children) [3]. The teams work in a transdisciplinary model in which professionals jointly plan and deliver interventions. Within the classroom context, this also involves teachers and classroom assistants. Overall, this approach aims to maximise clinical and cost effectiveness by enhancing the holistic nature of interventions and streamline the clinical pathway for children by professionals sharing their expertise [11].

In NI, children commence formal education at 4 years of age, and the mainstream school population includes a wide range of children including those with undiagnosed and diagnosed intellectual and/or developmental difficulties. The majority of children referred to the RISE teams are 4–5 year olds from schools in areas of SD. Attention and language difficulties are most frequently cited as the reason for referral [12]. Current support, developed and provided by the RISE teams, is a whole-class intervention targeting attention skills: the Attention and Listening Programme (ALP). This intervention has not been evaluated robustly and, unlike the new intervention developed in this study, it is not underpinned by working memory (WM) theory.

### Rationale for developing an intervention that targets WM

Working memory (the ability to hold in mind and mentally manipulate information over short periods of time in the face of distraction) is a cognitive skill linked to both everyday attentional skills and language development [13, 14]. Interventions aimed at improving WM may therefore enhance these closely related real-world skills [15]. However, the potential for WM interventions, and in particular computer-based training programmes, to improve untrained WM tasks and real-world skills (transfer effects) has been widely debated [16].

Overall, the evidence indicates that existing computer-based training programmes consistently produce gains on the trained tasks and closely related memory tasks [17, 18]. It has been suggested that to improve the therapeutic value of WM training, it may be necessary to embed it within typical classroom activities that are ecologically valid [19]. To test this, a new intervention was developed in the current study: the ‘Recall to Enhance Children’s Attention Language and Learning’ (RECALL) programme. This is a theoretically underpinned, evidence-based intervention that targets WM in 4–5-year-old children through group and whole-class activities over a 6-week period.

The experimental RECALL intervention is designed to be delivered by HPs from the RISE teams and teachers, and is co-produced with a group of these practitioners through a series of interactive workshops [20–22]. The intervention delivery model follows the current, collaborative practice of the RISE teams and schools in NI, where HPs model intervention sessions in the classroom so that teachers can observe the activities and strategies used and integrate them into their teaching. In this model, teachers are not trained directly in how to deliver classroom-based interventions, i.e., it is assumed that observing the HPs will be sufficient for them to replicate the intervention sessions. This has been suggested as a mutually beneficial aspect of collaborative practice in schools and a way of increasing intervention dosage for children at risk of language disorder within the constraints of the limited resource available for this work [23]. However, there is a lack of evidence to support this approach [3], so the feasibility of this model and how it may affect the fidelity of the intervention delivery was uncertain.

### Rationale for conducting a feasibility trial

Prior to conducting a definitive trial of RECALL, it was crucial to conduct a feasibility trial to deepen the understanding of the intervention [24] and to test whether it could be run with children as young as 4 years in a group setting with fidelity to its protocol. Previous evidence for classroom-based WM training interventions has been run with older children (6–7 year olds) who were trained on a one-to-one basis [25]. Little is currently known about the factors that might influence the implementation and dosage of classroom-based interventions for younger children with language disorder [26, 27]. The current study aimed to resolve the uncertainties about the feasibility of the experimental RECALL intervention and its delivery in a real-life context in which HPs model interventions for teachers. The existing collaborative practice between the RISE teams and schools in NI provided the optimal setting in which to investigate these issues.

### Study aims and objectives

This study aimed to determine the feasibility of conducting a definitive cluster randomised trial (CRT) evaluating whether RECALL is more effective than an existing intervention (ALP), and ‘education as usual’, in 4–5 year olds from areas of SD. The specific objectives of the study were as follows:

i) To examine the feasibility of the recruitment and sampling procedures.
ii) To measure the compliance and fidelity of the intervention delivery and explore the influence of the intervention delivery model on these factors.
iii) To explore the acceptability of RECALL to HPs and teachers who deliver classroom-based interventions in mainstream schools.
iv) To investigate the appropriateness of the outcome measures completed by teachers, children and their parents

## Methods

This section provides a summary of the study design, methods of the feasibility trial and the interventions implemented (RECALL and RISE). The study was designed and is reported according to the CONSORT 2010 extension to cluster randomised pilot and feasibility trials [28]. The trial was registered with the International Standard Randomised Controlled Trial Registry (ISRCTN13633886). Comprehensive details can be accessed in the study protocol associated with this trial [29].

### Study design

This was a three-arm, cluster randomised feasibility trial with a parallel group design that took place in two HSCT areas in NI. Two classes of 4–5 year olds were randomly allocated to each arm of the trial: (i) RECALL (experimental condition); (ii) the existing ALP intervention developed by the RISE teams (active control condition); and (iii) education as usual (no intervention condition). The experimental RECALL and active control interventions were delivered by HPs from the RISE teams once per week, followed up by two practice sessions delivered by teachers. Children’s outcomes were measured at baseline and 1-week post-intervention by research assistants (RAs). A process evaluation was conducted as part of the feasibility trial [30, 31]. This was based primarily on the framework for the design and reporting of process evaluations of cluster randomised trials [28]. It also included elements of Steckler and Linnan’s model [32] that are relevant to the delivery of classroom-based interventions. These included (i) the consideration of context (local factors that influence implementation); (ii) fidelity (the extent to which the intervention is delivered as conceived); (iii) the dose delivered (the amount of intervention offered to participants); and (iv) the dose accessed by individuals (the extent of participants’ engagement in the intervention).

### Recruitment and sampling

#### Target population

The target population were HPs from the RISE teams (SLTs, OTs, PTs and SEBs) who had experience of delivering classroom-based interventions and were not involved in the co-production of RECALL, mainstream primary schools situated in areas of SD based on data from the Northern Ireland Multiple Deprivation Measure (NIMDM) [33], teachers who had not previously received the ALP intervention and 4–5-year-old children.

#### Recruitment targets and sampling

As this was a feasibility study, a formal a priori power calculation was not conducted [34]. The results will not be used to estimate the sample size, intra-cluster correlation or treatment effects for a definitive trial because, in the case of cluster randomised feasibility trials, these can be unrealistic and misleading [35, 36]. Therefore, the recruitment targets in terms of clusters (schools) and individual participants were based on what was required to address the uncertainties about RECALL and its delivery in real-life contexts. The recruitment targets were eight HPs from the RISE teams and six schools. The aim was to recruit one class of 4–5 year olds in each school, with approximately 30 children in each class. In each class, the aim was to recruit a sample of 10 children in order to trial the outcome measures, meaning the total number of children involved in the study was 60.

To represent the typical range of ability in mainstream schools, a stratified sampling frame was used so that the sample of ten children from each class included three groups: (i) children about whom teachers have concerns around listening and communication skills but do not have a diagnosed developmental or intellectual difficulty (*n =* 5 per school, *n =* 30 in total); (ii) children with diagnosed developmental or intellectual difficulties (*n =* 2 per class, *n =* 12 in total); and (iii) typically developing children who do not have any identified listening and communication problems as recognised by the teachers *(n =* 3 per class, *n =* 18 in total). The proportion of children within each strata was determined on the basis of data regarding the incidence of special educational needs in mainstream schools in NI [37].

### Randomisation and blinding

Since the population of interest in this study is young children from areas of SD, it is possible that they may be in receipt of other interventions at the same time as RECALL. In a future full-scale trial of the effectiveness of RECALL, it will be vital to control for this confounding variable. Hence, there was a clear need to randomise the allocation of schools in this feasibility study. Randomisation took place at the school level after baseline data collection. The school names were placed in opaque envelopes that were randomly selected and allocated by the third investigator (overseen by the second investigator). The HPs were not blinded to the schools’ allocation as they inevitably knew which intervention they were delivering to which school. The school participants (principals, teachers and parents) and the RAs who conducted the outcome measurement with the children were blind to the allocation, i.e., they did not know which schools received the experimental RECALL intervention, the existing ALP intervention or education as usual (no intervention).

### Interventions

The experimental RECALL and active control (ALP) interventions were both 6-week interventions consisting of 40-min sessions repeated three times per week. Following the intervention delivery model typically employed by the RISE teams, the HPs modelled the first session each week for the teachers who provided two further practice sessions during the week (18 sessions in total). Details of these interventions are reported here according to the Template for Intervention Description and Replication (TIDieR) Checklist [38].

#### Experimental intervention: RECALL

This novel intervention targets WM explicitly and is based on a systematic review of evidence suggesting that repeated practice on certain (non-computerised) activities can improve WM and have the potential to produce effects on untrained WM skills (near-transfer) and real-world skills such as attention and language (far-transfer) [39]. The common ingredient across the effective interventions was the executive-loaded nature of the trained task, i.e., training on a task that taps into attentional and processing resources under executive control and not just the storage of information.

RECALL includes 3 executive-loaded tasks with specified dosage and task progression (

**Table 1:** RECALL components, dosage and task progression

**Executive-loaded task**	**Dosage**	**Task progression**
**Listening recall** [25] - Targets verbal ELWM. - The children listen to a short sentence, judge whether it is true or false, then recall the last word of the sentence	11 trials (practice items) per session.	The number of to-be-remembered words increases from one word in week one to two words by week 6.
**Odd one out** [25] - Targets verbal ELWM - The children look at three pictures in a grid, decide where the odd one out is (left, middle or right), then recall the location of the odd one out picture	11 trials per session.	The number of to-be-remembered locations increases from one in week one, to three or four by week 6.
**Phoneme awareness** [40, 41] - Targets the ability to isolate and manipulate sounds in spoken words, e.g., identifying the first sound in a word	10–15 min per session.	Difficulty increases from alliterative matching to blending onset and rime. Each task progresses from early to late developing phonemes based on typical speech sound development.

Table 1). Each session starts with a whole-class activity in which a fantastical theme is introduced for that week using a puppet, e.g., space. This is based on evidence that fantastical play supports children’s WM [42]. The class is then divided into 3 groups (of 9–10 children) that rotate around the three tasks, namely, listening recall, odd one out and phoneme awareness tasks (described in Table 1).

Evidence shows that the manipulation of WM loads on a trial-by-trial basis may be important for improving WM, i.e., training should be continually challenging (adaptive) [43]. Since RECALL is delivered to groups of children in the classroom, in the group context, individual adaptive profiles could not be rolled out. Instead, the trained tasks are designed to become progressively more difficult across its 6 weeks (see Table 1). Investigating the effectiveness of this approach will be a significant part of a full-scale CRT of RECALL, and capturing individual children’s responses as they complete the tasks will be important. Two methods of monitoring children’s progress from week to week have thus been integrated into the design of RECALL. For tasks that required a verbal response (listening recall and some of the phoneme awareness tasks), individual digital voice recorders were trialled with five children. For the odd one out task and the remaining phoneme awareness tasks, the children each had an individual booklet in which they marked their response using stampers.

To enable the HPs to deliver the intervention with fidelity to its protocol, they attended a 2-day training course prior to delivering RECALL that was facilitated by the first investigator. This covered the theoretical underpinning of RECALL in relation to WM and its associations with attention and language and also afforded the participants an opportunity to practise delivering the intervention tasks. The teachers were not provided with direct training on the intervention theory or delivery. This decision was based on two factors. Firstly, in the current collaborative practice of the RISE teams and schools, it is assumed that teachers will be able to implement classroom-based interventions having observed them in the classroom, without direct training. Mirroring current practice in this way makes this feasibility study pragmatic. Secondly, evidence from a qualitative study conducted prior to the intervention development indicated that, due to resource constraints, it was highly unlikely that teachers would be released from their everyday duties to attend training (paper in preparation).

Although direct training was not provided for the teachers, several steps were taken to support them in delivering the intervention delivery with fidelity. The RECALL package includes a comprehensive facilitators’ manual which covers the theory underpinning the intervention. It also has detailed session plans with scripted sections that the teachers can read out when introducing and delivering the intervention tasks. To make it easy for teachers to deliver RECALL, all of the resources required for each session are provided. Furthermore, to encourage the teachers’ compliance with the intervention delivery regarding the number of sessions provided, they were asked to complete a log in the RECALL manual to demonstrate that each session was delivered. Whether these steps were adequate (in the absence of direct training) to ensure a high level of compliance and fidelity is an issue that is investigated in this feasibility study, which could have significant implications for current practice.

#### Active control intervention: ALP

This pre-existing programme was informally developed by the RISE teams and aims to improve attention and listening skills through repeated practice of listening tasks and teaching children the importance of listening through visual and verbal cues. It is not underpinned by WM theory and does not require the children to recall verbal or visuospatial information.

#### No intervention control: education as usual

These schools did not receive any classroom-based interventions such as RECALL or ALP during the 6-week trial period.

### Outcomes

#### Primary outcome measures—feasibility

Table 2 provides an overview of the primary outcomes for this study, which relate to the feasibility of the trial processes and link directly to the four research objectives.

**Table 2:** Primary outcome measures—feasibility, data collected

**Research objective**	**Data collected**
To examine the feasibility of the recruitment and sampling procedures	The rates of actual recruitment compared to the recruitment targets were counted in terms of: • Number of schools (clusters) recruited • Number of HPs and teachers recruited to deliver the experimental and control interventions • Total number of children recruited for outcome measurement • Number and proportion of children recruited in each of the 3 subgroups within the stratified sampling frame
To measure the compliance and fidelity of the intervention delivery and explore the influence of the intervention delivery model on these factors	• Number and percentage of sessions delivered in each school • Structured observations of the delivery of RECALL in the classroom setting carried out by three of the investigators following Carroll et al. [44] (see Additional file 1) • Qualitative data from semi-structured interviews including reasons for any sessions not being completed (see Additional file 2)
To explore the acceptability of RECALL to HPs and teachers who deliver classroom-based interventions in mainstream schools.	• Measures of compliance and fidelity as an indication of acceptability • Semi-structured interviews • Comments on intervention logs
To investigate the appropriateness of the outcome measures completed by teachers, children and their parents	• The number and percentage of standardised assessments, teacher rating scales and parent rating scales completed pre- and post-intervention • The research assistants (RAs) report regarding the ease of administration and time taken to complete the battery of standardised assessments with the children

With regard to the measures of compliance and fidelity, observations of three RECALL sessions in each school (one delivered by the HPs and two by the teacher) were carried out by the first investigator. One session in each school was observed simultaneously and rated independently by the second or third investigator to reduce the risk that a single investigator’s judgement of fidelity could introduce bias to the study findings. The number of RECALL sessions observed in each school (three out of 18) equates to 16% of the total. This is consistent with suggestions in the literature that, for novel interventions, a minimum of 10% of their implementation should be observed [22]. Fidelity was scored using a structured checklist based on Carroll et al.’s 2007 framework (see Additional file 1) [44]. However, high fidelity was not determined on the basis of the scores alone, and there is no a priori defined score for this purpose. Rather, high or low fidelity was determined on the basis of whether the essential aspects of the proposed intervention components (in this case the WM load of the task) had been delivered in a way that RECALL may be effective. The scores were converted into percentages to allow comparison between the quality of the intervention delivery between the HPs and teachers and between schools in order to explore the factors that may influence the implementation of RECALL in real-life contexts. The fidelity data were also integrated with the findings from the semi-structured interviews with the HPs and teachers who delivered RECALL to determine the overall acceptability of intervention.

#### Secondary outcome measures—children’s WM, attention and language skills

The secondary outcome measures were standardised assessments of the children’s WM, attention and language completed at baseline and 1-week post-intervention. Following good practice in WM research [45], this included standardised assessment of: (i) the trained tasks (listening recall, odd one out and phoneme awareness); (ii) the untrained WM tasks (near-transfer); and (iii) attention and language skills (far-transfer effects). Table 3 details the assessments used. These were administered to determine the appropriateness of the outcome measures for a full-scale trial of RECALL (in line with the fourth research objective). The primary uncertainties related to the ease of administration and the time taken to complete a full battery of standardised assessments with 4–5 year olds (see Table 2). For these reasons, there were concerns around two measures in particular, i.e., the phoneme isolation subtest of the Preschool and Primary Inventory of Phonological Awareness (PIPA) [47] for phoneme awareness and the comprehension scale of the New Reynell Developmental Language Scales (NRDLS) [49] for language. Consequently, two alternative measures were trialled in one randomly selected school (*n =* 10 children): the phoneme segmentation subtest of the PIPA for phoneme awareness and the Clinical Evaluation of Language Fundamentals—Preschool (CELF-P) [50] for language. These two subtests were not included in the measures administered in the other 5 participating schools.

**Table 3:** Secondary outcome measures—children’s WM, attention and language skills

Outcome measured	Skill	Standardised assessment
**Trained task**	Trained WM tasks	**Automated Working Memory Assessment (AWMA)** **[**46**]** • A computerised assessment administered using a laptop. The children simply listen to automated instructions and provide a verbal or pointing response that is recorded by the facilitator. • 2 subtests administered in all 6 schools (*n =* 60 children): - Listening recall - Odd one out
**Trained task**	Phoneme awareness	**Preschool and Primary Inventory of Phonological Awareness (PIPA)** **[**47**]** • A standardised assessment consisting of 6 subtests for children aged 3 to 6 years 11 months • 2 subtests trialled: - Phoneme isolation subtest (administered in 5 schools, *n =* 50 children) - Phoneme segmentation subtest (administered in 1 school, *n =* 10 children)
**Near-transfer**	Untrained WM tasks	**Automated Working Memory Assessment (detailed above)** **[**46**]** • 4 further subtests administered in all 6 schools (*n =* 60 children): - Digit recall - Block recall - Counting recall - Non-word recall
**Far-transfer**	Attention	**A Developmental Neuropsychological Assessment (NEPSY-II) **[48] • Includes standardised performance-based measures of attention for children under 6 years • 2 subtests administered in all 6 schools (*n =* 60 children) - Auditory attention - Statue
	Language	**The New Reynell Developmental Language Scales (NRDLS)** [49**]** • A standardised assessment for children aged between 3 and 7 years 6 months. • Comprehension scale administered in 5 schools (*n =* 50 children) **Clinical Evaluation of Language Fundamentals—Preschool (CELF-P)** **[**50**]** • A standardised assessment for 3–6 year olds that examines children’s: understanding and use of syntax (grammar/sentence structure), semantics (word meanings) and grammatical morphology (markers of grammatical relationships • Core language subtests (*n =* 10) conducted in 1 school (*n =* 10)
	Behaviour in the classroom	**Behaviour Rating Scale of Executive-Function—Preschool Version (BRIEF-P)** **[**51] (*n =* 60) • A standardised, validated scale completed by teachers • Includes consisting of 63 items that can be used with children from 2 to 5 years 11 months to measure behavioural characteristics associated with executive function skills including WM • Completed by teachers in all 6 schools (*n =* 60 children)
	Communication skills at home	**Focus on Communication Outcomes Under Six–34 (FOCUS-34)** **[**52] **(*n =*** **60)** • A checklist of children’s communication skills at home completed by parents to measure change over time • Completed by parents in all 6 schools (*n =* 60 children)

### Data analysis

Regarding the primary outcomes, the feasibility data on recruitment rates, compliance and fidelity were analysed descriptively using means and percentages. The qualitative data collected via the semi-structured interviews with the HPs and teachers who delivered RECALL were audio-recorded, transcribed verbatim and analysed using Braun and Clarke’s [53] approach to thematic analysis. This analysis was underpinned by a realist research paradigm [54, 55] that informed every stage of the development of RECALL and the design and conduct of the current feasibility trial. The realist paradigm recognises the influence of context on the implementation and ultimate effectiveness of interventions in real-life settings [56].

The six phases of Braun and Clarke’s thematic analysis method [53] were followed to derive themes from the data in an inductive approach. To summarise the approach taken, each transcript was read, re-read and coded systematically with semantic codes, generated using the participants’ own words. The first investigator (A.R.) coded all of the transcripts, and the second investigator (J.T.) independently coded one complete transcript in a form of investigator triangulation [57]. The list of semantic codes was sorted into meaningful candidate themes, which were then depicted in visual representations (thematic maps) to examine the relationships between them. At this point, the initial list of codes was revisited, and the full data set was consulted again. The candidate themes were discussed on several occasions until set themes and sub-themes were agreed. Finally, during the writing up process, the wording of the themes was again revised by revisiting the full data set, initial codes and thematic maps in order to ensure the final interpretation of the data fully reflected the essence of the participants’ views.

Regarding the secondary outcomes, the data obtained from the standardised assessments completed with the children were not analyses for statistical significance of treatment effects because this study was under-powered for this purpose.

### Ethical approval

Ethical approval was granted by the Ulster University Research Ethics Committee (REC/18/0036), and approval was obtained from the relevant HSCT research offices.

## Results

This section presents the results of the study for the four research objectives regarding the feasibility of the recruitment and sampling procedures, the compliance and fidelity of the intervention delivery, the acceptability of RECALL to HPs and teachers and the appropriateness of the outcome measures completed by teachers, children and their parents.

### Recruitment and sampling

#### Recruitment rates

**Fig. 1:**
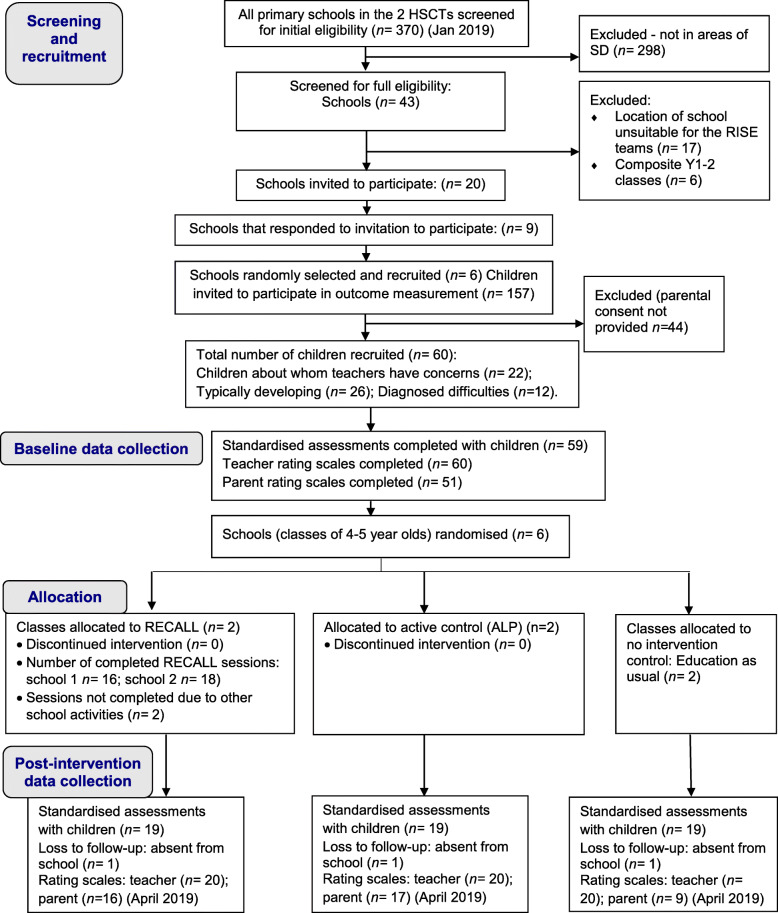
RECALL cluster randomised feasibility trial flow chart (following CONSORT guidance, 2010) [28]

Figure 1 shows the CONSORT flow chart [28] including the response, recruitment and retention rates throughout the study. It shows that recruitment targets were met in terms of the total number of HPs (*n =* 8), schools (*n =* 6) and children (*n =* 60). After the exclusion criteria were applied, 10 schools were invited to participate in the study, and 9 responded. Of these, 6 schools were randomly selected to take part. In terms of retention, no schools or individual participants dropped out of the study.

#### Sampling

Table 4 provides details of the numbers and characteristics of the schools (clusters) and the individual participants recruited to the study compared to the recruitment targets. This shows that, whilst the overall recruitment targets were achieved, some aspects of the recruitment process did not achieve their aim. Due to staff absence (maternity leave/sick leave), the RISE teams could only facilitate the study in particular geographical sectors within their HSCT areas. Consequently, from the list of schools identified in areas of SD (*n =* 43), a considerable number (*n =* 17) had to be excluded on the basis of their location. As a result, the criteria in respect of SD were widened to include schools ranked within the lowest quintile within the HSCT (rather than the lowest decile). The overall rate of parental consent (72%) was good. However, some parents of children about whom teachers had concerns did not consent, and the desired proportion of children in this sub-group was not achieved (*n =* 22, 37% compared to the target of *n =* 30, 50%). It was also apparent during the sampling process that teachers did not always know whether children did/did not have a diagnosis. Two children did not complete post-intervention assessments as they were absent from school, indicating minimal loss to follow-up (3%).

**Table 4:** Participant characteristics

Participants	Recruitment targets	Number recruited	Characteristics
Health professionals	*n =* 8	*n =* 8	Professional backgound: SLT (*n =* 4), OT (*n =* 2), PT (*n* = 1), SEB (*n* = 1)
Schools (clusters)	*n =* 6	*n =* 6	Social disadvantage ranking (based on data from the NIMDM 2017 [33]): Within lowest decile for their HSCT area (*n =* 3) Within lowest quintile for their HSCT area (*n =* 3)
Children recruited for outcome measurement	*n =* 60	*n =* 60	Gender: girls (*n =* 26, 43%); boys (*n =* 34, 57%) Age at baseline, 56 to 67 months (mean = 61 months)
	*n =* 30 (50% of sample)	*n =* 22 (37%)	1) Children about whom teachers had concerns around listening and communication skills
	*n =* 12 (20%)	*n =* 12 (20%)	2) Children with diagnosed developmental or learning difficulties
	*n =* 18 (30%)	*n =* 26 (43%)	3) Typically developing children who did not have any identified listening and communication problems as recognised by the teachers

### Compliance and fidelity to the intervention delivery

The data gathered through the activity logs completed by teachers in their RECALL manuals indicated that there was good compliance with the intervention delivery regarding the total number of sessions completed (95%) and the number of trials delivered (11 practice items of listening recall and odd one out, and 10–15 min of phoneme awareness training). In terms of the quality of delivery, for the RECALL sessions delivered by the HPs, there was a good degree of fidelity to the intervention protocol (76%). For the teacher-delivered sessions, fidelity varied between the 2 schools (there was a high degree of inter-rater consistency on the fidelity measure across the research team): school 1 (76%) and school 2 (45%). The observation data collected during the school visits and the qualitative data from the semi-structured interviews revealed that this discrepancy related to the delivery of the odd one out task during the teacher-delivered RECALL sessions. In school 1, the teacher divided the class into three groups, as specified in the intervention protocol. In school 2, the teacher presented the task to all of the children at the same time, holding up the picture stimuli and walking around the classroom until each child had seen them. Then, the children all stamped the location of the odd one out picture in their booklets. The interview carried out with this teacher revealed that she changed the task in this way because she prefers to work with the whole class together. The result was that it lengthened the time that the children had to hold the information in their WM, both changing the nature of the task and making it too difficult. The overall duration of the session also increased, and the children, especially those who were inattentive, became unmotivated and restless.

### The acceptability of RECALL

**Fig. 2:**
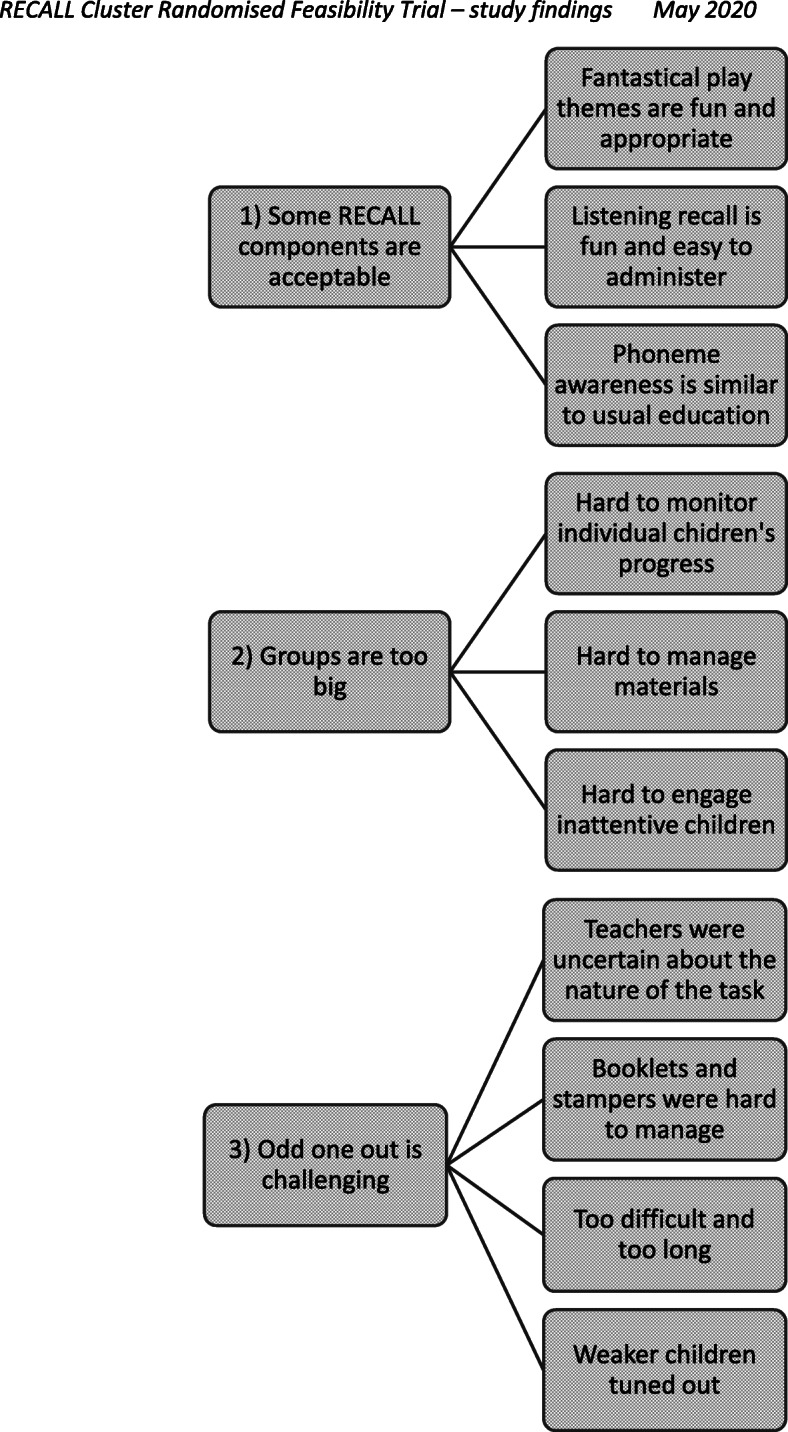
Qualitative data themes identified in semi-structured interviews with the HPs and teachers who delivered RECALL

The data gathered through the observations of RECALL in the classroom and via the semi-structured interviews with the HPs and teachers indicated mixed findings in relation to the acceptability Figure 2 shows that three major themes were identified through the integration of these data. Each theme is described below along with some exemplar quotes from participants.

#### Theme 1: some RECALL components are acceptable

All of the HPs and teachers liked the fantastical play component of RECALL, reporting that the puppet, fantastical themes and props were appropriate and fun for 4–5-year-old children. The phoneme awareness tasks were easy to administer due to their similarity to usual classroom practice. The listening recall task was also quick and easy to administer. It was at an appropriate level of difficulty (with both the teachers and the HPs reporting that the children seemed to improve across the 6-week intervention period) and engaged the class. The fact that the sentences tied in with the fantastical themes and were funny seemed to appeal to even the most inattentive children. One of the teachers reported:

I think, the listening recall one benefitted and involved every child…..It was actually boys I noticed who probably stick out with the listening recall and the boys who like imaginative play and who like a giggle. So, I actually found that really related to boys. It related to everybody, but they stood out. It surprised me that they were interested. It was just because they thought it was funny, so it just hooked them in and they wanted to be part of it.

The use of individual digital voice recorders (to monitor the children’s performance on the listening recall and some of the phoneme awareness tasks) was impractical. The devices used were acceptable to the HPs and teachers because they were unobtrusive and did not interfere with the delivery of the task. However, the microphones picked up too much background noise from the classroom, meaning the child’s voice could not be distinguished. It was also difficult to hear the facilitator’s voice when presenting the trial items, so the accuracy of the child’s response could not be judged.

#### Theme 2: odd one out is challenging

None of the HPs or teachers liked the odd one out task in its current format. The teachers were uncertain about the nature of this specific task and how to deliver it, e.g., whether it was acceptable for children to place their fingers on the location of the odd one out picture in their booklets. The children needed help to turn the pages of the booklets. Many of the children were distracted by the stampers and tended to stamp ad hoc in their booklets. To manage this, the HPs or teachers had to repeatedly pause the task to ensure all of the children were on the right page. This disrupted the flow of the activity and elongated it, meaning that the task could not be delivered in a way that would be effective, and the data gathered in the booklets were unreliable.

In addition, the participants all reported that there were too many trial items per session so the children became unmotivated, especially those with existing attention difficulties who tended to copy their peers’ responses. The HPs and teachers all stated that the difficulty level increased too quickly and the children would have benefitted from additional practice at the 2-to-be-remembered item level. One of the teachers stated:

I found it was a very big challenge for a lot of them [the children]. At the start it wasn’t too bad, but then as it progressed and maybe you were at three odd one out on the one page, then four- it was really, really difficult. Again, those few [children] in the top group would have been trying to focus really well but so many just lost it and a lot of them were randomly stamping. The wee weaker groups, they just weren’t focused at all.

#### Theme 3: groups are too big

Whilst the use of booklets and stampers to record children’s responses impacted on the acceptability of RECALL, the size of the groups was also identified as a barrier to the intervention delivery. The number of children in the class (divided into groups of 9 or 10) made it difficult to deliver the tasks and to monitor children’s progress. This was noted by all of the HPs and teachers during the semi-structured interviews (even for the listening recall task which was universally liked by the participants).

This was summed up by one of the HPs:

…..if there was less children it would be so much easier to guide and judge how they were doing. Because you were only getting a general idea [of how they were doing].

### The appropriateness of the outcome measures

As stated earlier, a future large-scale trial of the effectiveness of RECALL would have to include a range of measures of children’s outcomes including measures of the trained activities (WM and phoneme awareness skills), untrained WM tasks (near-transfer) and attention and language skills (far-transfer effects) [45] (Table 3). The primary uncertainties here related to the ease of administration and the time taken to complete a full battery of standardised assessments with 4–5 year olds (see Table 2).

Overall, the rate of completion at the post-intervention time point shows that 95% of the standardised assessments were completed with the children (see Fig. 1). However, reports from the RAs who conducted the outcome measurement indicated that administering the full battery of assessments with each child was time-consuming (on average more than 1 h per child). This may have impacted negatively on the children’s motivation and performance. In particular, the New Reynell Developmental Language Scales (NRDLS) took a considerable amount of time to complete, whereas the Clinical Evaluation of Language Fundamentals—Preschool (CELF-P) [50] (trialled in one school for comparison) was much quicker to administer. With regard to the auditory attention and statue subtests of the Developmental Neuropsychological Assessment (NEPSY-II) [48], all of the RAs found it difficult to observe and simultaneously record the children’s performance. Therefore, they doubted the accuracy of their scoring. If this test were used in a full trial, thorough training and practice should be provided to those administering it, and inter-rater reliability must be measured.

Regarding the proxy measures of children’s functional skills, 100% of the teacher rating scales of attention in the classroom were completed at both time-points (pre- and post-intervention) using the Behaviour Rating Scale of Executive Function—Preschool Version (BRIEF-P) [51]. This suggests that the checklist was acceptable to teachers. Children’s communication skills at home were measured using the Focus on Communication Outcomes Under Six–34 (FOCUS-34) [52]. This tool looks at change/improvement in the child’s communication skills over time (rather than providing a direct measure of their ability). It should be completed by the same parent at each time point with support from a SLT [52]. Due to the classroom-based nature of this trial, the forms were sent home for parents to complete and return to the school. Therefore, the parents completed this measure without support. Completed checklists were returned at both time points for 35 children (58% of the sample), but examination of the raw data indicated that for 8 children the forms were not completed by the same parent at the two time points. This raises questions about the reliability of the data. Furthermore, two outlying scores were apparent indicating possible misunderstanding of scoring (a Likert scale) by the parents. In a future trial, greater support would need to be provided to parents (as outlined in the protocol for the FOCUS-34) to avoid these potential issues.

## Discussion

This study responds to calls for globally significant, rigorous and ecologically valid research into collaborative, classroom-based approaches for children at risk of language disorders and the factors that may impact on their delivery [3–7]. To our knowledge, RECALL is the first theoretically underpinned, evidence-based, classroom-based intervention that specifically targets WM to enhance attention and language skills in 4–5-year-old children from areas of SD.

The overall research aim was to determine whether it is possible to conduct a definitive CRT to evaluate whether RECALL is more effective than an existing intervention (ALP) and education as usual. The successful recruitment of HPs, schools and children from areas of SD; high completion rates; and minimal loss to follow-up suggest that the trial processes could be scaled-up into a definitive trial. However, because staffing levels within the RISE teams may fluctuate, consultation with the service managers will be essential for the successful roll out of a large-scale study. With regard to the generalisability of the study findings to areas in the UK or beyond that do not have support equivalent to the RISE teams, it is envisioned that RECALL could be delivered by any HPs working within school-based services, where the provision of classroom-based support has become a routine aspect of practice [3].

The stratified sampling method employed in the current study should be modified because this was affected by ambiguity around whether some children had a diagnosis or not. Therefore, in a large trial, the three strata could be collapsed into two: typically developing children and those about whom teachers have concerns and may/may not have a diagnosis. In this study, there was an under-representation of children with developmental difficulties, which seemed to be related to parents not consenting to participation (rather than small number of children in these classes with difficulties). This is a potential source of bias that must be addressed in a larger trial. Engagement with key stakeholders was an important facet of the development of RECALL and the feasibility of carrying out the current study. Further consultation with professionals (HPs, teachers, school principals and other representatives from the education sector) will be carried out prior to the roll out of a larger trial to identify ways of increasing parental consent rates for this population.

Including children considered to be typically developing would be valuable in a large trial since high proportions of children in areas of SD are at risk of language disorders, and little is known about the individual differences that moderate the effects of WM training [58] and language interventions [59]. A full trial will require a large sample with sufficient power to detect differences between subgroups of children as well as intervention groups [35].

The second research objective was to measure the compliance and fidelity to the intervention protocol. Overall, the compliance levels were good with the vast majority of the intervention sessions (95%), suggesting it would be feasible to conduct a full-scale trial of RECALL. The high level of fidelity to the intervention protocol during the RECALL sessions delivered by the HPs (76%) (that would have been higher if the odd one out task had been easier to deliver) also supports the concept of conducting a full trial. Furthermore, it suggests that 2 days of training adequately enables HPs to deliver RECALL. Regarding the teacher-delivered sessions, the inconsistency and variation in fidelity between the two teachers' delvery of RECALL highlight the importance of facilitator factors in intervention delivery. The teachers’ uncertainty about how to deliver the tasks demonstrates that the detailed intervention manual and demonstration provided by the trained HPs were not sufficient. This supports the provision of direct training on the theoretical underpinning and delivery of RECALL for all teachers involved in a future definitive trial of the intervention. This should include a minimum of 8 h direct instruction, as well as coaching and feedback [3]. Teachers could also be given a video of the tasks being demonstrated accurately that they could refer to as required to support their implementation in the classroom. These measures would better ensure fidelity of task delivery which is essential for the therapeutic effectiveness of the intervention, particularly the odd-one-out task (and was lost for half of the participants in the RECALL arm of this trial).

Exploration of the acceptability of RECALL produced mixed findings. The HPs and teachers liked the listening recall and fantastical play components. These were considered to be fun and at an appropriate level for 4–5 year olds. The phoneme awareness component was also acceptable. The fact that the teachers reported that these tasks are similar to their usual practice may suggest they are not required in RECALL. However, since the descriptive analysis of the post-intervention scores showed a trend towards improvement for the intervention groups, further investigation of the effectiveness of these tasks could be valuable in a full-scale trial.

The odd one out task is not acceptable to HPs or teachers in its current form for two key reasons. First, they found it difficult to manage the materials, which included picture stimuli, booklets and stampers. Second, many of the children became inattentive during this task. These factors relate to the task itself, but also to the classroom setting and the children’s characteristics. The task delivery could be simplified by enabling the children to indicate the odd one out location by pointing. The dose delivered per session (11 practice items) and dose frequency (three times per week) may also have been too intense for 4–5 year olds. In addition, from week three onwards, the task became too difficult, i.e., there were too many items to be remembered. These findings are consistent with emerging evidence regarding the effects of dosage on the outcomes of language interventions, suggesting that if treatment is too intense it can be detrimental to children’s learning [5, 26, 59]. The current study underscores the need for robust investigation of dosage in both WM and language interventions. Modifications to RECALL, including the task delivery and its dosage, could be explored through further co-production work and small group work with 4–5 year olds prior to a full-scale trial.

In relation to the classroom setting and the children’s characteristics, the potential effectiveness of RECALL was impeded by the size of the groups set up for the task (9–10 children) and their composition, where the weaker children were observed copying their more-able peers. Many of the children, particularly the most at risk children (already presenting with inattentive behaviour in the classroom) became unmotivated by a task that was too challenging for them.

**Fig. 3:**
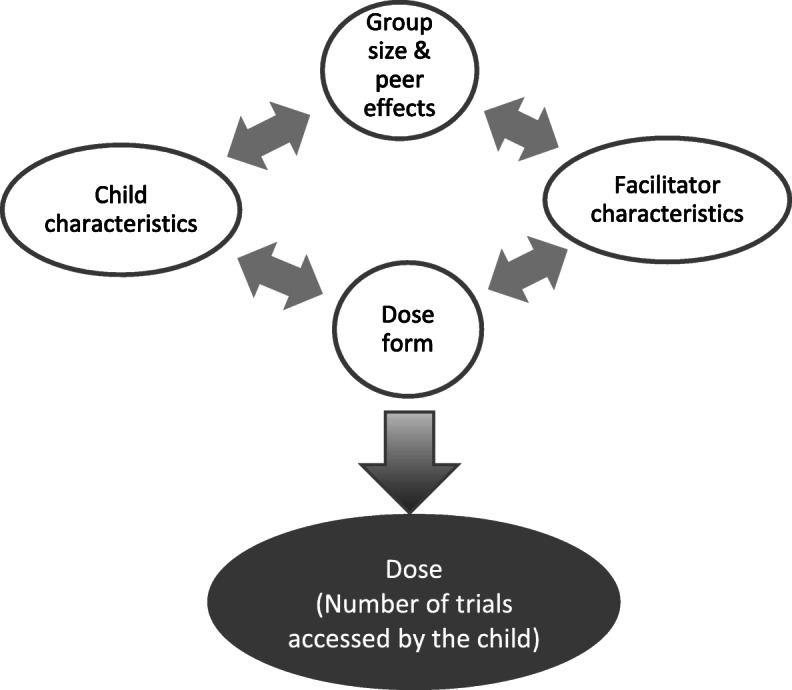
The factors impacting on dosage in classroom-based interventions

The evidence discussed so far has illuminated a dynamic interplay between the way a therapeutic task is presented and its difficulty level (dose form), the setting within which it is delivered such as group size, children’s characteristics including their motivation and attention and facilitator characteristics, e.g., theoretical knowledge of the task and how to deliver it and personal preference in terms of teaching methods. This complex blend impacts on and can dilute the number of trials accessed by individual children, particularly those who are most at risk of language disorder. Figure 3 graphically represents this.

Regarding the appropriateness of the pre-and post-intervention outcome measures, the descriptive statistics suggest that the following measures could be used in a full trial: the AWMA for working memory, the phoneme isolation subtest of the PIPA for phoneme awareness, the BRIEF-P and FOCUS-34 (completed according to the protocol) as proxy ratings of attention in the classroom and communication skills at home and the NEPSY-II for attention (provided thorough training is provided for those administering it and the inter-rater reliability is assessed). The phoneme segmentation subtest of the PIPA could be used in addition to the phoneme isolation subtest. This would add minimal time to the assessment process, and its inclusion would mitigate against any risk of reduced sensitivity of the phoneme isolation subtest, which many of the children performed well on in the current study. To assess language, the NRDLS should be replaced by the CELF-P since this takes less time to administer and should be more acceptable for both the child and RA administering it. The use of digital voice recorders to monitor verbal responses is not feasible in the classroom setting, and the use of booklets impeded the completion of the odd one out task. Therefore, alternative methods of monitoring children’s performance on a weekly basis, perhaps by a trained observer, will be required in a definitive trial.

### Limitations of the present study

This was a small scale study with just two schools in each arm of the trial. However, the findings are strengthened by the study design through the inclusion of an active control group receiving an intervention of comparable structure and dosage to the experimental RECALL intervention. The need to widen the school eligibility criteria to include those in the lowest quintile of SD means that half the sample were in less disadvantaged areas than originally anticipated. This raises questions about whether the findings are generalisable to schools in more disadvantaged areas of NI or beyond. In addition, compliance and fidelity to the delivery of the active control intervention was not measured and this should be addressed in a definitive trial.

## Conclusions

RECALL is a novel, multi-component intervention that targets WM to enhance attention and language skills. To our knowledge, this is the first paper to report on the feasibility of implementing a WM intervention in real-life contexts. With the exception of the methods used to monitor children’s progress from week to week, the trial processes could be scaled-up into a future definitive trial to evaluate the effectiveness of RECALL.

In relation to the intervention components, this study has provided unique evidence of the potential effectiveness of the two directly trained WM tasks (listening recall and odd one out) for children as young as 4–5 years. Listening recall was implemented successfully and was acceptable to the HPs and teachers who delivered the intervention; and odd one out could be modified to enhance its acceptability and the fidelity of its delivery.

Overall, the potential effectiveness of RECALL for the children who may benefit most from it (i.e., those presenting as inattentive in the classroom and are at risk of low WM) could be optimised if it were implemented in small group settings. This would enhance its acceptability to HPs and teachers and improve its potential effectiveness by maximising the dosage accessed by individual children. RECALL could be modified through further co-production work and feasibility testing involving small group work with 4–5 year olds. This study has highlighted the challenges of balancing empirically evidenced dosage with the feasibility and acceptability of what can be delivered in real-life contexts. Furthermore, it emphasises the need for teachers to have thorough training on the theoretical underpinning to interventions for children with language disorders in the mainstream classroom.

## Supplementary Information

**Additional file 1:** Checklist for fidelity of intervention delivery.

**Additional file 2:** Schedule for post-intervention semi-structured interview.

## Data Availability

As this is a feasibility trial, sharing of the dataset is not anticipated. However, any requests for data or material should be made to the corresponding author. Requests will be reviewed by the Trial Steering Committee.

## Abbreviations

ALP: Attention and Listening Programme
AWMA: Automated Working Memory Assessment
BRIEF-P: Behaviour Rating of Executive Functions—Preschool
CA: Classroom assistant
CELF-P: Clinical Evaluation of Language Fundamentals—Preschool
CRT: Cluster randomised trial
ELWM: Executive-loaded working memory
FOCUS-34: Focus on Communication Skills Under Six-34
HSCT: Health and Social Care Trust
NEPSY-II: A Developmental Neuropsychological Assessment
NI: Northern Ireland
NRDLS: New Reynell Developmental Language
OT: Occupational therapist
PIPA: Preschool Inventory of Phonological Awareness
PT: Physiotherapist
RA: Research assistant
RISE: Regional Integrated Support for Education
RECALL: Recall to Enhance Children’s Attention, Language and Learning programme
SD: Social disadvantage
SEB: Social, emotional and behaviour specialist
SLT: Speech and language therapist
STM: Short-term memory
WM: Working memory
