# Supplementary material for: A classroom intervention targeting working memory, attention and language skills: a cluster randomised feasibility trial
Source: Pilot Feasibility Stud. 2021 Feb 6;7:45. doi: 10.1186/s40814-021-00771-w (PMC7866677; doi:10.1186/s40814-021-00771-w)
Supplement: Supplementary file 1 — Additional file 1: Checklist for fidelity of intervention delivery. [file 40814_2021_771_MOESM1_ESM.docx]

**Additional file 1: RECALL Feasibility study - Checklist for fidelity of intervention delivery**

| **RECALL session observed (1- 6): _________________________**  **Programme facilitated by: RISE Team professional/Teacher (delete as appropriate)**    **Date of observation: ___________ Rated by (research team member): ­­­­­­­­­­­­­­­­­­­_____________** | | | |
| --- | --- | --- | --- |
| **Element** | **Criteria** | **Rating**  1 = low and 9= high.  Circle as appropriate | **Comments** |
| Content | The executive-loaded nature of the trained tasks was maintained. | 1 2 3 4 5 6 7 8 9 |  |
|  | All of the tasks in the session plan were delivered. | 1 2 3 4 5 6 7 8 9 |  |
|  | Session delivered by RISE NI Team only – use of auditory recording | 1 2 3 4 5 6 7 8 9 |  |
| Coverage | The specified number of trials were administered per activity (dose) | 1 2 3 4 5 6 7 8 9 |  |
| Frequency | All of the children participated in each activity | 1 2 3 4 5 6 7 8 9 |  |
| Duration | The session lasted 40 minutes. | 1 2 3 4 5 6 7 8 9 |  |
|  | Each activity was presented within its suggested timeframe. | 1 2 3 4 5 6 7 8 9 |  |
| Intervention complexity | The resources were manageable. | 1 2 3 4 5 6 7 8 9 |  |
|  | The tasks appeared to be at an appropriate level for the children. | 1 2 3 4 5 6 7 8 9 |  |
| Facilitation strategies | The programme manual was referred to during the session. | 1 2 3 4 5 6 7 8 9 |  |
| Quality of delivery | The introductions/instructions to activities were given as specified in the programme manual. | 1 2 3 4 5 6 7 8 9 |  |
|  | The facilitator presented the session in an engaging way for the children. | 1 2 3 4 5 6 7 8 9 |  |
|  | Tasks were suitably differentiated for individual children. | 1 2 3 4 5 6 7 8 9 |  |
| Participant responsive-ness. | The teacher and classroom assistant were engaged in the session. | 1 2 3 4 5 6 7 8 9 |  |
|  | The children found the activities engaging. | 1 2 3 4 5 6 7 8 9 |  |
| **Questions raised by participants:** | | | |
| **Please note any guidance provided by research team:** | | | |
| **Please note any additional barriers or facilitators to the programme implementation:** | | | |
| **Any other comments/observations:** | | | |

Reference: Based on a conceptual framework for implementation fidelity (Carroll *et al*. 2007) [44]
