# Supplementary material for: A classroom intervention targeting working memory, attention and language skills: a cluster randomised feasibility trial
Source: Pilot Feasibility Stud. 2021 Feb 6;7:45. doi: 10.1186/s40814-021-00771-w (PMC7866677; doi:10.1186/s40814-021-00771-w)
Supplement: Supplementary file 2 — Additional file 2: Schedule for post-intervention semi-structured interview. [file 40814_2021_771_MOESM2_ESM.docx]

**Additional file 2: Schedule for post-intervention semi-structured interview**

| **Introduction** |
| --- |
| Use introductory script to explain the purpose of the interview participants’ role in the study and assure participants of confidentiality and anonymity. |
| **Exploring acceptability of the research processes** |
| For RISE NI team members – How did you feel about the random allocation of schools?  For teachers:  How did you feel about being randomly allocated to a group?  How did you feel about the selection of children for outcome measurement?  How easy/challenging was the process of gaining parental consent?  *Prompts for discussion: ratio of children in each group. Would they have liked more specific criteria for selection?* |
| **Exploring participants’ experience of delivering the RECALL** |
| **Programme content and resources**  What do you think about the use of the Memory Mack puppet?  What do you think of the fantastical themes?  What do you think about the 3 RECALL tasks?   - Odd one out - Listening Recall - Phoneme awareness   What do you think of the resources provided for these tasks? E.g., size, quality  Did you refer to the program manual? If so, how often?  How helpful is the manual?  What do you think of the programme structure (working in small groups)  **Dosage**  How easy was it for you to incorporate the program into your everyday work?  What do you think about:   - the number of practice trials of each activity? - the number of sessions per week?   For RISE NI team members - were you able to deliver all six sessions?  For teachers- were you able to provide two follow-up sessions per week?  What factors impacted on this?  **Difficulty level**  What do you think of the difficulty level for each task specified in the program?  Were you able to monitor the children’s progress from week to week?  Do you think the children’s skills improved?  **Overall**  What activities did you like?  What activities did you not like?  How did the children respond to the program?  How effective did you feel the program was?  *Prompts for discussion:*   - *If people find it difficult to recall specific activities – use each session plan to jog their memory.* - *Can you tell me why you liked/did not like that activity? What was it about the task that made it difficult?*   *What makes you say it was effective/ineffective?* |
| **Exploring acceptability of the program and the outcome measures** |
| How easy/difficult was it to use the digital voice recorders?  For the teachers:  What are your thoughts on completing the BRIEF-P?  How easy was it to complete?  Did it add to your understanding of children’s skills?  How likely do you think it is that other teachers would complete it? |
| **Exploring barriers and facilitators to implementation in large scale RCT and longer-term practice** |
| If you had access to RECALL, how likely is it that you would use it again? (use in full/use parts?)  If unlikely to, how could it be altered so that you would want to use it?  If you had to use the program again, how could this be made easier for you?  Is there anything that would make it difficult for other health professionals/teachers to deliver the program?  What resources/training/support would you need to use the program again?  *Prompts for discussion:*   - *Explore factors at personal, intrapersonal and organisational level*   *Discuss group size, adult support in the classroom* |
| **Close**   - Provide brief summary of information gathered. - Check if participants would like to make any additional comments - Thank participants for their time |
